# Re-visiting the evolution, dispersal and epidemiology of Zika virus in Asia

**DOI:** 10.1038/s41426-018-0082-5

**Published:** 2018-05-09

**Authors:** John H.-O. Pettersson, Jon Bohlin, Myrielle Dupont-Rouzeyrol, Ola B. Brynildsrud, Kristian Alfsnes, Van-Mai Cao-Lormeau, Michael W. Gaunt, Andrew K. Falconar, Xavier de Lamballerie, Vegard Eldholm, Didier Musso, Ernest A. Gould

**Affiliations:** 1 Infectious Disease Control and Environmental Health, Norwegian Institute of Public Health, Oslo, Norway; 2 Marie Bashir Institute for Infectious Diseases and Biosecurity, Charles Perkins Centre, School of Life and Environmental Sciences and Sydney Medical School, University of Sydney, Sydney, NSW 2006 Australia; 3 Department of Microbiology, National Veterinary Institute, Uppsala, Sweden; 4 Department of Medical Biochemistry and Microbiology (IMBIM), Zoonosis Science Center, Uppsala University, Uppsala, Sweden; 5 Unité de Recherche et d’Expertise–Dengue et autres Arboviroses, Institut Pasteur de Nouvelle-Calédonie, Noumea, New Caledonia; 6 Unit of Emerging Infectious Diseases, Institute Louis Malarde, Papeete, Tahiti French Polynesia; 7 IRD, AP-HM, SSA, VITROME, IHU-Méditerranée Infection, Aix Marseille Université, Marseille, France; 8 London School of Hygiene and Tropical Medicine, London, UK; 9 Departmento de Medicina, Universidad del Norte, Barranquilla, Colombia; 10 UMR “Unité des Virus Emergents”, Aix-Marseille Université–IRD 190–Inserm 1207–IHU Méditerranée Infection, Marseille, France; 11 APHM Public Hospitals of Marseille, Institut Hospitalo-Universitaire Méditerranée Infection, Marseille, France

## Abstract

Based on serological evidence and viral isolation, Zika virus (ZIKV) has circulated for many years relatively benignly in a sylvatic cycle in Africa and an urban cycle in South East Asia (SEA). With the recent availability of limited but novel Indian ZIKV sequences to add to the plethora of SEA sequences, we traced the phylogenetic history and spatio-temporal dispersal pattern of ZIKV in Asia prior to its explosive emergence in the Pacific region and the Americas. These analyses demonstrated that the introduction and dispersal of ZIKV on the Pacific islands were preceded by an extended period of relatively silent transmission in SEA, enabling the virus to expand geographically and evolve adaptively before its unanticipated introduction to immunologically naive populations on the Pacific islands and in the Americas. Our findings reveal new features of the evolution and dispersal of this intriguing virus and may benefit future disease control strategies.

## Introduction

Zika virus (ZIKV) was first isolated in 1947 from rhesus monkey serum collected in the Zika Forest of Uganda (Africa)^1^. Serosurveys conducted from the 1950s onwards suggested that ZIKV circulated in several countries of Africa and Asia. Less than 20 human infections were confirmed within the first 60 years following the discovery of the virus, probably because it causes mild disease in humans and is often misdiagnosed given the existence of several clinically similar diseases, for example, dengue or chikungunya fever^2, 3^. ZIKV emerged in Africa (African lineage) and subsequently spread through Asia (Asian Lineage). In 2007, an Asian lineage strain of ZIKV unpredictably caused a unique explosive epidemic on the Pacific island of Yap in Micronesia^4^, involving 70% of the population. However, within months, the epidemic subsided, and there has only been one further report of epidemic ZIKV in Micronesia in the Kosrae State in 2016^5^. However, in October 2013, a more recent ZIKV strain emerged in French Polynesia (FP)^6^, probably following the accumulation of functionally important amino acid substitutions^7, 8^, which were predicted to enhance virus transmission by the primary vector species *Aedes aegypti*^7^. Subsequently, non-vector borne transmission (mother to child, sexual and blood transfusion transmission) was confirmed with the emergence of ZIKV in the Americas^2, 9^. Following its establishment on the FP islands, ZIKV radiated eastwards and westwards^10^ and subsequently emerged in Brazil before dispersing and causing epidemics in the Americas^2, 11^.

Many new sequences are now available from ZIKV-infected humans in Asia, including Bangladesh^12^, India^13^, Indonesia^14^, the Philippines^15^, Singapore^16^, Thailand^17^, and Vietnam^18^. These sequences may impact our interpretation of the early evolutionary history of ZIKV in Asia. We therefore re-visited the evolutionary events of ZIKV from its first emergence in Asia. Although additional sequence data will expand our knowledge, it is already possible with the new data from India and South-East Asia (SEA) to interpret the dispersal patterns of ZIKV in terms of its geographic source, mode of spread, and time of circulation.

Using updated phylogeographic and phylogenetic analyses, we illustrate and discuss how the epidemic pattern in FP and the Americas contrasts strongly with the relatively long-term and apparently benign circulation of ZIKV in SEA.

## Results

**Fig. 1:**
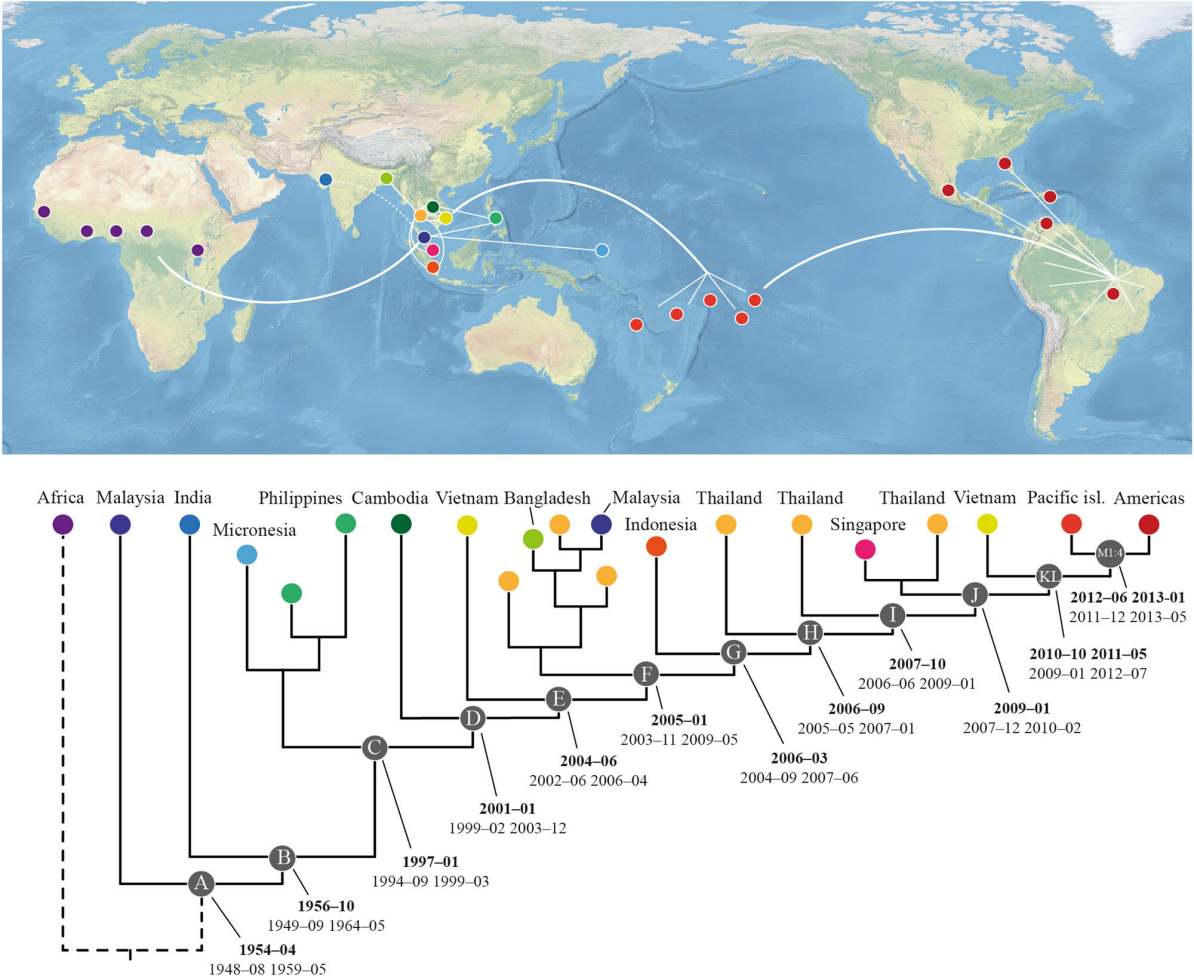
Illustrative phylogenetic tree of ZIKV evolution and dispersal in the Asian region. Coloured circles on the terminal nodes represent the country of infection on the map. White lines indicate the main routes of spread. Letters within the gray circles (A–M4) refer to diversification events throughout the period of ZIKV evolution in Asia and the Pacific islands, which correspond to the dates of divergence (in bold) and highest posterior density for each tMRCA. A comprehensive tree is provided in the Supplementary Materials (Suppl. fig. S5)

Bayesian phylogenetic analysis of ZIKV sequences from Africa and Asia, including partial data from a recently available Indian ZIKV sequence^13^, indicate that the Indian virus shares a more recent ancestry with all of the other Asian sequences than with the Malaysian sequence, which is positioned as a sister-group to all of the other Asian sequences (Fig. 1, branch leading to node B, Posterior probability 0.71; Suppl. fig. S1). We also performed separate Bayesian analyses of both the partial capsid and NS2b/NS3-genes (Suppl. fig. S2 and S3, respectively). Our results demonstrated that Indian ZIKV shared a more recent ancestry with the SEA viruses compared to the Malaysian virus. However, a tree based on a partial envelope gene sequence placed the Indian virus in the most basal position of the Asian ZIKV clade (Suppl. fig. S4). Whether this truly represents the phylogenetic position of Indian ZIKV will require a complete genomic analysis of the isolate and more sequence data from geographically distant regions of India and neighbouring countries. Subsequently, and separated by a long branch, ZIKV then diverged to form a clade that harboured the majority of Asian ZIKV diversity (Fig 1, node C). This was followed by a series of diversification events (Fig. 1, nodes C–L) that predominantly occurred in SEA and were characterized by frequent introductions and localized outbreaks that probably reflected the increasing distribution and spread of ZIKV in the primary vector species, i.e., *Ae aegypti*, and susceptible humans. Notably, both Thailand and Vietnam experienced introductions and outbreaks of multiple ZIKV strains throughout the course of its evolution in SEA and have served as sources of ZIKV in other regions (Fig. 1, Suppl. fig. S1 and S5).

An inclusive phylogenetic and temporal analysis, focusing on isolates positioned after the dispersal of ZIKV from Africa but prior to its emergence on the Pacific islands and the Americas, was prepared. The evolutionary rate of the Asian ZIKV lineage under a strict clock was estimated to be 7.26 × 10^−4^ substitutions per site per year (95% HPD: 6.28–8.19 × 10^−4^). Furthermore, the analysis showed that the most recent common ancestor (MRCA) of ZIKV in Asia (Fig. 1, node A) was present 5–12 years earlier than previously estimated^7, 19^, ~1954–04 (95% highest posterior density: 1948–08 to 1959–05). Then, ~2 years later, circa 1956–10 (95% HPD: 1949–09 to 1964–05), the MRCA of the Indian lineage and all subsequent Asian lineage ZIKV emerged (Fig. 1, node B), surprisingly implying the introduction of Indian ZIKV from an Asian rather than an African source. Subsequently, following a silent period of ~40 years, ZIKV appeared in SEA in early 1997 (95% HPD: 1994–09 to 1999–03; Fig. 1, node C). It should also be noted that the MRCA of the Micronesian and Philippine sequences was estimated to have been present in Asia ca. 1997 (95% HPD: 1995–07 to 1999–12; Suppl. fig. S5). Thus, the ancestor of these two emergent viruses was possibly present in SEA and evolved relatively silently for at least 10 years prior to the Micronesian epidemic in 2007. Following the emergence of ZIKV in SEA (node C), there was a period of circulation of ~15 years (Fig. 1, nodes C–L) prior to its introduction and emergence on the FP islands (Fig. 1, nodes M1–M4) in mid-2012 (95% HPD: 2011–12 to 2013–01). The introduction of ZIKV from FP into America followed about 1 year later, ~2013–03 (95% HPD: 2012–11 to 2013–06) (Suppl. table 1; Suppl. fig. S5). When the Indian sequence data were excluded, the results of the temporal estimates were confirmed by using the HPD values that overlapped in the two analyses, which were supported by time-regression analyses including all data (Suppl. fig. S5–S7).

## Discussion

Following its first isolation in April 1947 from a sentinel rhesus monkey in the canopy of the African Zika Forest of Uganda^1^, ZIKV was isolated again in 1948 in the same forest but this time from *Ae africanus*, an indigenous African sylvatic mosquito^1^. These observations suggest that in Africa, ZIKV circulates between sylvatic *Aedes* species mosquitoes and monkeys that inhabit the forest canopy, which does not exclude other possible transmission cycles. Subsequent epidemiological and serological studies have revealed that ZIKV was probably also prevalent and widespread in African towns and villages during the 1950s^2, 20, 21^. Since it is now known that both dengue viruses (DENVs) and ZIKV adapted from sylvatic to urban transmission cycles, likely due to the increasing *Ae aegypti* population density in urban districts of SEA, our observations provide a rational explanation for how it was possible for ZIKV to be isolated in 1966 from domestic *Ae aegypti* in populous areas of the Malaysian peninsula^22^. Importantly, attempts in the 1950s to isolate ZIKV from a wide range of mosquitoes in sylvatic environments in Malaysia^22^ and other Asian countries did not result in the identification of additional strains of ZIKV, implying that the Malaysian virus was not significantly established in the sylvatic Asian environment, which also appears to be the case for ZIKV in the Pacific and the Americas; that is, ZIKV is not significantly sylvatic except in Africa. Nevertheless, following the detection of ZIKV in Malaysia, subsequent serosurveys conducted between 1952 and 1989, in which 189 of 1011 tests were reported as ZIKV positive^2, 23–25^, support the concept of widespread distribution of ZIKV in SEA^2, 19, 26–29^. Importantly, these early studies were performed using the most specific serological assay, viz., neutralization against high virus challenge doses (>50 LD_50_ to >1000 LD_50_) in mice, to reveal a high incidence of neutralizing antibody responses in residents of Malaya (19% and 75%)^23, 25^, Borneo (18%)^23^ and India (16.8%)^24^.

Why did ZIKV disperse so successfully throughout Asia for so many years without appearing to have caused major epidemics^30^? Clearly, the very low frequency of clinically apparent ZIKV infections and the difficulty in clinically discriminating between infections caused by DENV, chikungunya virus (CHIKV), and ZIKV were major factors in the failure to recognize the presence of ZIKV infections. Second, the inapparent spread of endemic ZIKV throughout SEA resulted in the emergence of low-level herd immunity to the virus. Third, antigenically related flaviviruses, including DENV, West Nile virus (WNV), Japanese encephalitis virus, Wesselsbron virus, and Stratford virus, circulating throughout SEA contributed a background of cross-protective reactivity to ZIKV that might have dampened ZIKV transmission. By contrast, with the exception of several islands in the Western Pacific region that have experienced outbreaks of JEV, DENV was the only human pathogenic flavivirus known to have caused outbreaks in islands further east in the Pacific prior to the arrival of epidemic ZIKV. Moreover, it is a widely held view that immunity to DENV alone does not protect humans against infection with ZIKV^2^. Thus, the evolving ZIKV was probably introduced to populations of immunologically naive inhabitants on most of the Pacific islands, leading to the explosive epidemics that have now been reported widely^7^. Several human flaviviruses, including DENVs, are known to circulate in the Americas. Thus, the situation in Brazil and neighbouring South American countries should differ significantly from that described in Oceania because several human pathogenic ZIKV-related flaviviruses circulate in Brazil, including DENVs, St. Louis encephalitis virus (SLEV), yellow fever virus (YFV), Ilheus virus (ILHV), Rocio virus (ROCV), and others. Why, therefore, was ZIKV able to cause explosive epidemics in the Americas in the presence of this potential immune background? The most likely explanation is that, with the exception of YFV, which circulates primarily in the sylvatic environment in the Americas, and DENV, which is not believed to stimulate protective immunity to ZIKV^2^, these other ZIKV-related flaviviruses are not commonly transmitted by domestic *Ae aegypti* and therefore do not circulate primarily in the urban environment favored by ZIKV.

Moreover, a serosurvey of Indian human sera using the mouse protection test revealed a high incidence of protective immunity against DENV-1 (40.4%: 72/196), DENV-2 (20.2%: 38/188), WNV (35.0%: 123/351), and ZIKV (16.8%: 33/196), while none of 588 sera neutralized YFV and only a few neutralized the American flaviviruses, ILHV (1.4%: 3/206) or SLEV (2.8%: 6/211)^24^. These results suggest that the presence of an antibody-mediated cross-protection between ZIKV and either YFV or the American flaviviruses is relatively unlikely. In addition, while ROCV caused local epidemics in coastal areas of São Paulo, Brazil from 1973 to 1980, it has subsequently disappeared from this region of Brazil^30^. Furthermore, WNV, which has only recently been detected in Brazil, does not yet appear to have become established as a significant pathogen in Latin America^31^. It should also be emphasized that even following the epidemics in the Pacific and subsequent spread of ZIKV to the Americas, Asia has experienced relatively small ZIKV outbreaks compared to the explosive and clinically apparent epidemics in the Pacific and the Americas^32^.

Thus, ZIKV epidemics in FP and the Americas have occurred in the presence of a high background immunity to DENV. However, as noted above, it is a widely held view that immunity to DENV does not necessarily protect humans against infection with ZIKV^2^. How and when did African ZIKV gain access to SEA? Like DENV and CHIKV, the major transmission vector of ZIKV is *Ae aegypti*, the domestic descendant of the sylvatic African *Ae africanus*^2, 33^ and the primary conduit for the introduction of DENV and CHIKV to Asia via ships trading out of East Africa during the 18th–20th centuries^34–36^. Thus, by analogy, ZIKV is also likely to have been introduced to Asia, from Africa, in infected humans and/or *Ae aegypti*, to which ZIKV had adapted, via the same shipping routes. This argument is also supported by contemporary evidence of frequently reported cases of ZIKV being introduced to non-tropical countries by infected individuals returning from areas in the tropics where epidemic ZIKV was known to be circulating^37^. Based on an earlier study^7^ with a similar estimated evolutionary rate (Median: 6.30 × 10^−4^; 95% HPD: 5.48 × 10^−4^ to 7.10 × 10^−4^) to that of the present study, the African and Asian ZIKV lineages were estimated to have diverged in ~1834, which is consistent with reports of other *Ae aegypti-* transmitted viruses, namely, DENV and CHIKV, being introduced via ships trading between East Africa through India to SEA^34, 38^.

Recently, ZIKV was isolated from a patient in northern India. A neighbour-joining analysis of the partial envelope gene of this virus implied that the Indian sequence was basal in the Asian ZIKV clade^13^. To investigate this further, we concatenated sequences of the partial capsid, envelope and NS2b/NS3 genes of the Indian ZIKV isolate and analyzed the data in a Bayesian phylogenetic framework. Our results suggested that the Indian sequence was not basal to the Malaysian virus isolated in 1966 (Suppl. fig. S1). By contrast, our analysis suggests that ZIKV could have been introduced to India from SEA, rather than vice versa. Although the currently available sequence data do not robustly resolve the Indian versus Malaysian ancestry, the concept of the introduction of ZIKV from SEA to India seems plausible given that ZIKV also appears to have been introduced to Bangladesh from SEA (Fig. 1; Suppl. fig. S1 and S5). In this context, the highest incidence of human sera that neutralized ZIKV was collected from towns in the Ahmedabad district (north-west India) in the state from which the NIV1720741 strain used in the present analysis was isolated in 2016^24^. This state may, therefore, represent a pocket of endemicity containing divergent ZIKV strains in that region.

Our phylogenetic and temporal analyses suggest that ZIKV was first introduced to SEA during the 1950s, and then dispersed both eastwards and westwards ~5–12 years earlier than was previously predicted^7, 19^. Our estimate is also compatible with serological studies conducted in Malaysia, India, Indonesia, the Philippines, Thailand and Vietnam, indicating that ZIKV was present in these countries between 1952 and 1954^2^ (and references therein). Based on the current phylogenies (Fig. 1; Suppl. fig. S1 and S5), ZIKV was potentially first introduced from Africa to the Malaysian–Indonesian region and subsequently to India. However, although many new sequences have been published during the past few years, it is still not possible to determine precisely whether any particular country in SEA has played a major role compared with other countries in the evolution and dispersal of ZIKV in SEA.

Between 1954 and 1976, no recorded human clinical ZIKV cases in Asia were confirmed using serological tests or virus isolation procedures. Hence, it is likely that ZIKV remained undetected because it was primarily a sub-clinical disease and diagnostic tests for ZIKV were not routinely performed. Then, in 1977 and 1978 three and four febrile patients on Java, Indonesia, were diagnosed with ZIKV infection, respectively^39^. Over 35 years later, ZIKV was isolated from a patient from Sumatra, Indonesia^14^. It is possible that ZIKV remained undetected in the Malaysian–Indonesian region for a long time, circulating mainly between *Ae aegypti* and human hosts, causing sporadic sub-clinical or mildly clinical human infections, while evolving and adapting to its vector species. Thus, since the late 1950s, ZIKV appears to have circulated silently in SEA according to serosurvey data. The analyses suggest that the MRCA of the Malaysian and Indian isolates is approximately 40 years earlier than that of the Micronesian/Philippine and all subsequent isolates (Fig. 1, node B vs C, Suppl. fig. S5). However, without further sequence data from these neglected areas of Asia, it will continue to be difficult to identify precisely where ZIKV was circulating and, potentially, causing human ZIKV infections. Nevertheless, serological studies suggest that ZIKV may have been present in the Indian–SEA region earlier than the 1950s^23, 25, 40^.

It is clear that ZIKV has circulated without recognition for many years in Asia. This is supported by evidence from serology, virus isolation^2^ and phylogenetic analyses, all of which have shown that the ancestral Asian virus continued to evolve and circulate (Suppl. fig. S1 and S5) prior to and after its unexpected emergence as an epidemic virus on Yap island, Micronesia, in 2007^4^. In fact, during a period of 14 years, 1997–2011 (Fig. 1, nodes C–L), this ancestral lineage of Asian ZIKV appears to have dispersed between several other countries in SEA. Notably, Thailand and Vietnam were possibly the source of ZIKV in other regions of Asia. For example, Thailand appears to have been the source of ZIKV in Singapore, which experienced a large outbreak in 2016^16^, and in Bangladesh (Suppl. fig S5). Thus, it is likely that different countries in SEA have experienced different epidemiological situations. Whereas, the Philippines seems to have had a single strain of ZIKV circulating for several years, Thailand and Vietnam appear to have experienced multiple introductions and “waves” of ZIKV that have swept across these countries (Suppl. fig S1 and S5) followed by re-occurring outbreaks^29, 41^. Consequently, following the isolated Micronesian epidemic in 2007, ZIKV continued to circulate and evolve in the SEA–Indian-region^32, 42^ before causing a large-scale epidemic in FP in 2013/2014. ZIKV was subsequently introduced into the Americas, apparently via Brazil^7, 8, 11^.

It is also believed that the ZIKV strain that emerged on Yap Island, Micronesia, did not disperse widely beyond Micronesia. Although the reasons for its failure to spread have not been identified, there are several possible explanations. First, the Micronesian lineage pre-dates the FP lineage by at least six years, during which four unique and functionally significant amino acid substitutions emerged in the FP lineage, i.e., T777M, V763M, S139N, and M/T2634V^7^. The first three of these unique substitutions has been shown to have functions relating either to receptor attachment or virus fusion activity with cell membranes^43^. These substitutions could therefore have an impact on the relative transmission efficiency of the FP lineage compared with earlier Asian lineage sequences^7^. Second, in support of this interpretation, the unique S139N substitution, first identified in 2016^7^, emerged in FP and was detected in virtually every descendant lineage, suggesting a possible link with microcephaly. Subsequently, the unique S139N substitution was associated with increased ZIKV infectivity in humans and enhanced fetal mouse microcephaly^44^. Third, the relatively small human population size and density on the Micronesian islands and limited connectivity with other Pacific islands might have limited the likelihood of ZIKV dispersal to other distant regions of the Pacific. Fourth, a mutation (A188V) was subsequently shown to potentially contribute to the apparently increased transmission efficiency of ZIKV^45^. However, more than one amino acid substitution appears to determine the transmission efficiency since the A188V substitution is not consistently associated with it. For example, A188V is the ancestral state for this described substitution in Africa, but the only outbreak reported in Africa was caused by a strain belonging to the Asian lineage^46^. Nevertheless, the data, in total, are consistent with the hypothesis that ZIKV has circulated, potentially silently in the presence of other related human pathogenic flaviviruses, in sub-Saharan Africa and SEA for many decades^47–49^.

Our analysis also indicates that the MRCA of the lineage that eventually led to the large outbreak on FP might have existed and potentially been introduced in June 2012 (95% HPD: 2011–12 to 2013–01), i.e., ~1 year before the first detection of ZIKV in FP in October 2013^6, 9, 50, 51^. Based on the observed short divergence times between nodes M1 and N (Suppl. table 1; Suppl. fig. S5), a potential explanation for the rapid dispersal of ZIKV in the Pacific and the Americas could be interpreted as being due to relatively low levels of herd immunity in the populations. In addition, a similar cryptic period of about 1 year, before its appearance as an epidemic virus, was predicted for the introduction of ZIKV into Brazil^11^. Whether this reflects the relatively mild nature of ZIKV infections and consequently low viraemic levels in the majority of the infected population is an intriguing subject for mathematical modelers.

In summary, following its emergence in Asia, after being introduced from Africa, ZIKV circulated as a relatively benign virus in Asia for several decades. ZIKV then emerged in Oceania and the Americas and, largely because of its association with central nervous system malformations in neonates, especially microcephaly, it became an international public health concern. This raises at least two important questions, (i) how did ZIKV circulate successfully throughout Asia during its apparent silent period between 1966 and 2006 and (ii) what are the major determinants of the increased epidemicity of ZIKV in the Pacific and the Americas. With regard to the former question, several possible explanations have been proposed for these differences in the epidemicity of ZIKV in Asia compared to that of ZIKV in the Pacific and the Americas: Stochastic evolution^52^, adaptive evolution^7^, NS1 protein enhancement^45^, S139 enhancement^7, 44^, and contrasting levels of background immunity due to cross-protective antibodies^2^. Each of these factors could contribute to the overall differences in the transmission efficiency. However, the relatively high background cross-reactive antigenicity and/or immunity, which are likely present in Asia, contrasting with the relatively low equivalent antigenicity and/or immunity in the Pacific and the Americas, in combination with the key amino acid mutations in the ZIKV genome, can potentially explain the different epidemiological patterns between Asia on the one hand and the Pacific and Americas on the other.

## Materials and methods

Partial and complete genome ZIKV sequences were retrieved from the NCBI GenBank (www.ncbi.nlm.nih.gov/genbank/) focusing on Asian lineage ZIKV isolates. Initially, all available complete genomes (as of October 2017) were retrieved. Sequences collected from the Pacific islands and the Americas were subsampled to reduce the data load. Subsequently, partial phylogenetically informative sequences were collected around the Indian–SEA region and added to the dataset. These sequences were then used to construct two curated open reading-frame datasets (sequence information is available in Supplementary Table S2); (i) one open reading-frame dataset included 89 sequences, representing 5 from the African lineage and 84 of the Asian lineage; (ii) the other open reading-frame dataset included 84 sequences of Asian lineage only. The datasets were aligned with Mafft v.7.266^53^, keeping the reading-frame consistent with amino-acid positions, and were visualized and edited in AliView^54^. All subsequent analyses were performed with a generalized time-reversible nucleotide substitution model with four gamma distributed rate variation categories and a proportion of invariant sites, as selected by jModeltest v.2^55^.

To analyze the phylogenies of the 84 Asian lineage strains, including the novel Indian sequence, a Bayesian phylogenetic tree was computed using MrBayes v.3.2.6^56^ with the dataset including the 5 African ZIKV lineage strains as an out-group. Two parallel runs with four Metropolis-coupled chains were initiated for 5 M Markov chain Monte Carlo generations using the previously determined models of nucleotide evolution with default flat Dirichlet priors, sampling every 1000 generations and discarding the first 25% as burn-in before computing a consensus tree. Since only partial sequences of the Indian isolate were available, it is likely that phylogenetic placement was also examined by constructing three separate trees based on either the capsid (453 bp), envelope (773 bp), or NS2b/NS3 (1393 bp) sequence alignments.

To estimate the evolutionary rates and time to the most recent common ancestor for the Asian lineage, BEAST v.1.8.3^57^ was employed. Initially, the temporal structure was assessed with TempEst V.1.5.1 (http://tree.bio.ed.ac.uk/software/tempest/) plus MM-type robust regression^58^. Subsequently, a path-stone and stepping-stone model-test was performed^59^, determining that a strict molecular clock with a non-informative continuous-time Markov chain (CTMC) prior and a Bayesian skyline coalescent tree prior with a piecewise-constant demographic model best suited the dataset based on Bayes factor evaluation. Based on the resultant Bayesian phylogenetic tree, which included African ZIKV as outgroup sequences, all Asian ZIKV lineages except the Malaysian 1966-sequence were annotated as a monophyletic clade. The robustness of the dating was also evaluated by excluding the Indian sequence. Using the models and parameters suggested by the model-test, two analyses were run in parallel for 100 M MCMC generations, sampling every 10,000 generations per analysis. The convergence of the two runs was assessed with Tracer 1.6 (http://tree.bio.ed.ac.uk/software/tracer/). Tree- and log-files were combined with LogCombiner (BEAST-package^57^), and a maximum-clade credibility tree was computed with TreeAnnotator (BEAST-package^57^) after discarding the first 10 M MCMC generations of each run. The resultant consensus tree was visualized and edited in FigTree v.1.4.1 (http://tree.bio.ed.ac.uk/software/figtree/). All computations were run using the CIPRES computational cluster^60^.

## Electronic supplementary material

Supplementary figure 1

Supplementary figure 2

Supplementary figure 3

Supplementary figure 4

Supplementary figure 5

Supplementary figure 6

Supplementary figure 7

Supplementary table 1

Supplementary table 2
