# Supplementary material for: Re-visiting the evolution, dispersal and epidemiology of Zika virus in Asia
Source: Emerg Microbes Infect. 2018 May 9;7:79. doi: 10.1038/s41426-018-0082-5 (PMC5940881; doi:10.1038/s41426-018-0082-5)

**Supplementary figure S1 – Phylogenetic tree of Zika virus including African and Asian isolates based on partial and complete genomes**

The tree was computed with MrBayes using a dataset that included 5 African lineage ZIKV isolates as an outgroup in addition to 84 Asian lineage ZIKV sequences. Two parallel analyses with four MCMC chains were run for 5M MCMC generations using a GTR+I+G(4) model of nucleotide evolution, Dirichlet priors, sampling every 1000 generations, discarding the first 25 % as burn-in before computing a consensus tree. Numbers on branches indicate posterior probability support.

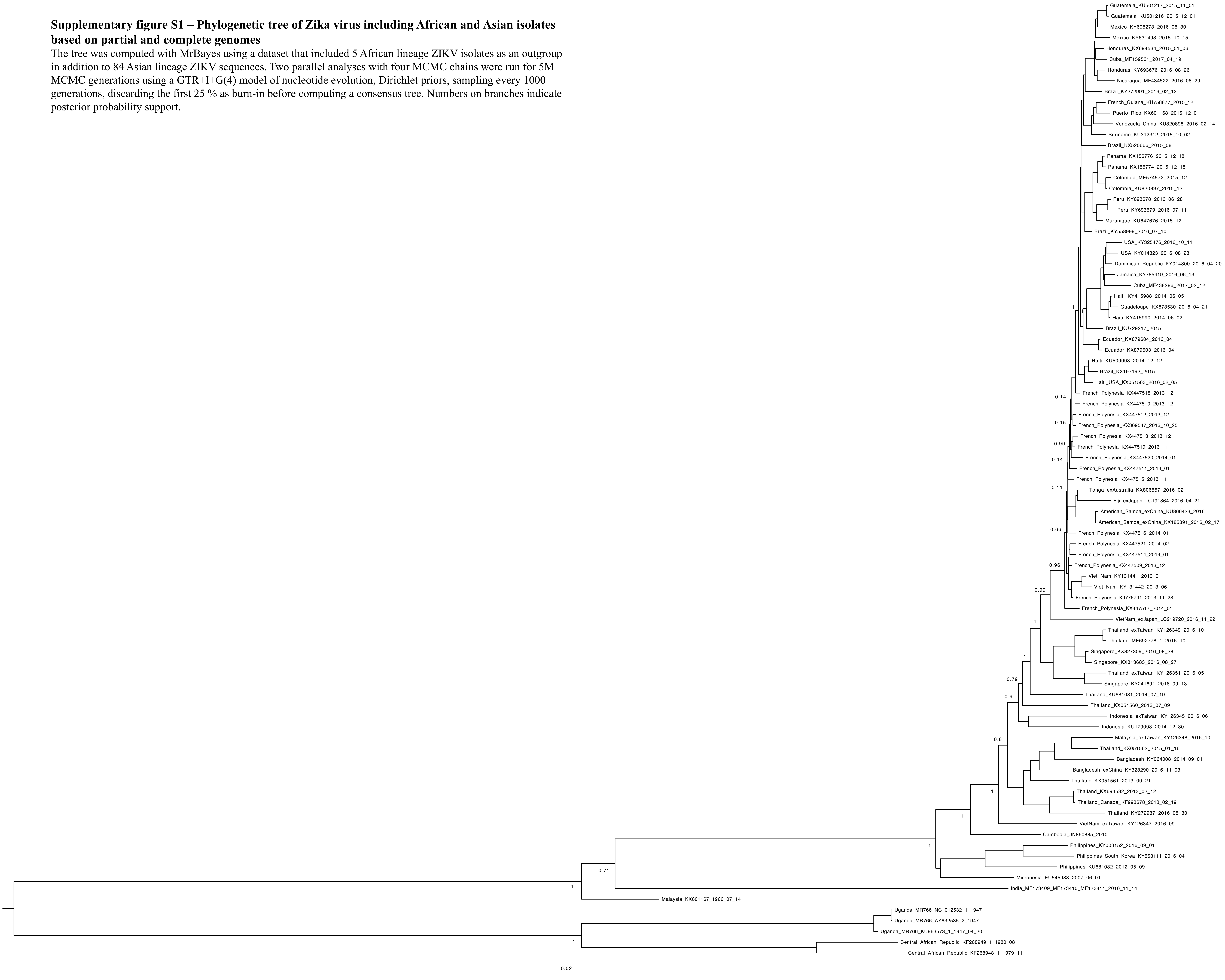

Supplement: Supplementary file 1 — Supplementary figure 1 [file 41426_2018_82_MOESM1_ESM.pdf]
