# Supplementary material for: Re-visiting the evolution, dispersal and epidemiology of Zika virus in Asia
Source: Emerg Microbes Infect. 2018 May 9;7:79. doi: 10.1038/s41426-018-0082-5 (PMC5940881; doi:10.1038/s41426-018-0082-5)

Supplementary figure S2 – Phylogenetic tree of Zika virus including African and Asian isolates based on the partial capsid gene (453bp).

The tree was computed using MrBayes with a dataset that included 5 African lineage ZIKV isolates as an outgroup in addition to 82 Asian lineage ZIKV sequences (two partial Asian ZIKV sequences were removed due to missing data). Two parallel runs with four MCMC chains were run for 5M MCMC generations using the GTR+I+G(4) model of nucleotide evolution, Dirichlet priors, sampling every 1000 generations, discarding the first 25 % as burn-in before computing a consensus tree. Numbers on branches indicate posterior probability support.

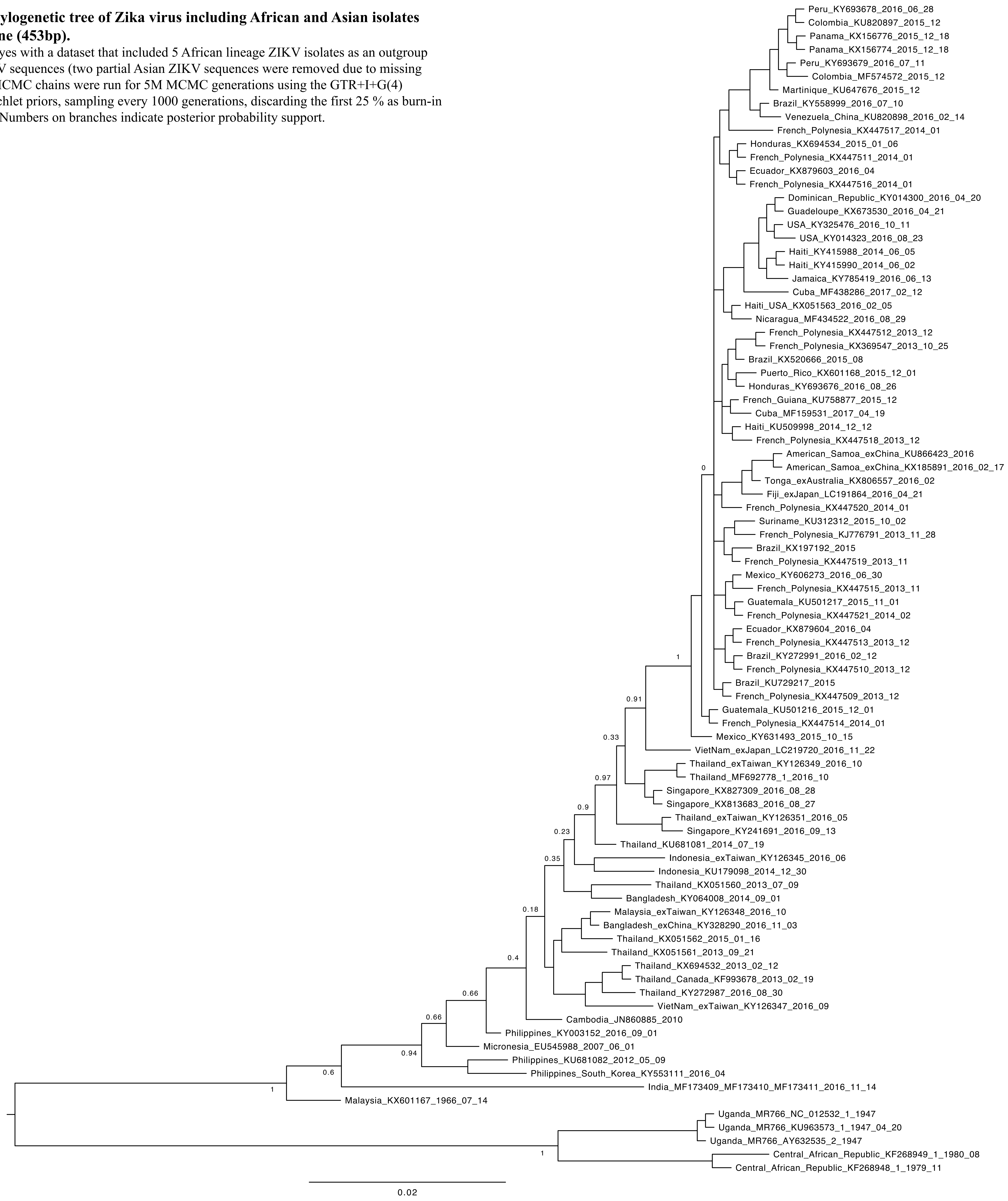

Supplement: Supplementary file 2 — Supplementary figure 2 [file 41426_2018_82_MOESM2_ESM.pdf]
