# Supplementary material for: Re-visiting the evolution, dispersal and epidemiology of Zika virus in Asia
Source: Emerg Microbes Infect. 2018 May 9;7:79. doi: 10.1038/s41426-018-0082-5 (PMC5940881; doi:10.1038/s41426-018-0082-5)

Supplementary figure S3 – Phylogenetic tree of Zika virus including African and Asian isolates based on the partial NS2b/NS3 genes (1393bp).

Phylogenetic tree computed using MrBayes with a dataset that included 5 African lineage ZIKV isolates as an outgroup in addition to 76 Asian lineage ZIKV sequences (eight partial Asian ZIKV sequences were removed due to missing data). Two parallel analyses with four MCMC chains were run for 5M MCMC generations using a GTR+I+G(4) model of nucleotide evolution, Dirichlet priors, sampling every 1000 generations, discarding the first 25 % as burn-in before computing a consensus tree. Numbers on branches indicate posterior probability support.

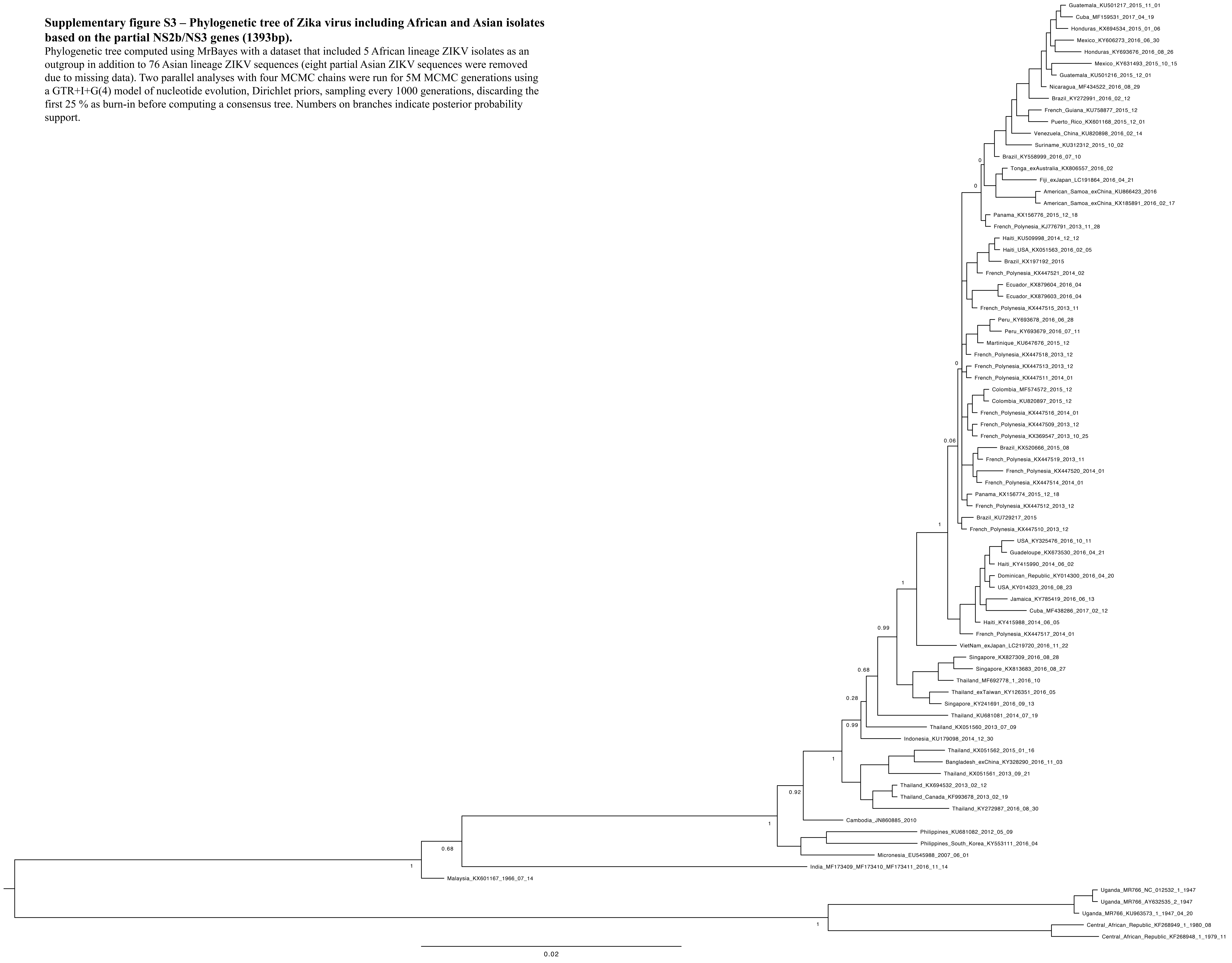

Supplement: Supplementary file 3 — Supplementary figure 3 [file 41426_2018_82_MOESM3_ESM.pdf]
