# Supplementary material for: Re-visiting the evolution, dispersal and epidemiology of Zika virus in Asia
Source: Emerg Microbes Infect. 2018 May 9;7:79. doi: 10.1038/s41426-018-0082-5 (PMC5940881; doi:10.1038/s41426-018-0082-5)

Supplementary figure S5 – Complete and partial genome Asian ZIKV tree dated with BEAST

Evolutionary rates and time to most recent common ancestor for 84 complete and partial genome Asian lineage sequences were estimated with BEAST v.1.8.3 (see methods section in main text also). Following assessment of temporal structure with TempEst v.1.5.1 and Bayes factor modeltest, a strict molecular clock with a non-informative CTMC prior, and a Bayesian skyline coalescent tree prior, with a piecewise-constant demographic model, was employed on a dataset with 84 Asian lineage sequences. After burn-in, the final tree was summarized as a maximum clade credibility tree. Numbers on nodes indicate tMRCA and 95% HPD as years in the past from 2017-04-19.

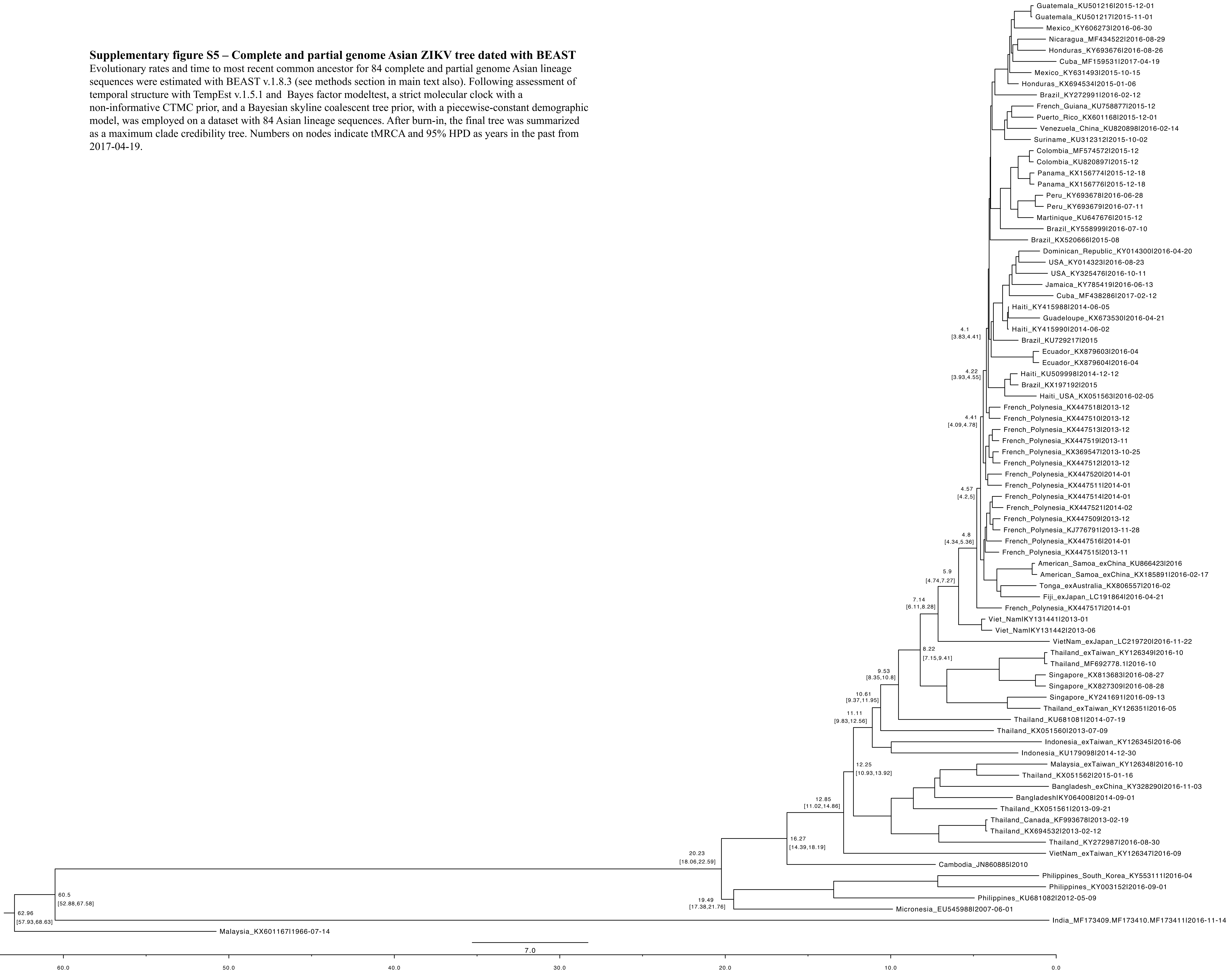

Supplement: Supplementary file 5 — Supplementary figure 5 [file 41426_2018_82_MOESM5_ESM.pdf]
