# Supplementary material for: Re-visiting the evolution, dispersal and epidemiology of Zika virus in Asia
Source: Emerg Microbes Infect. 2018 May 9;7:79. doi: 10.1038/s41426-018-0082-5 (PMC5940881; doi:10.1038/s41426-018-0082-5)

Supplementary figure S7 – TempEst and MM regression of Asian ZIKV

Regressions were made with TempEst v.1.5.1. (A) and with MM regression (B) using robust regression (blue line) and ordinary least squared regression (OLS, black dashed line).

A

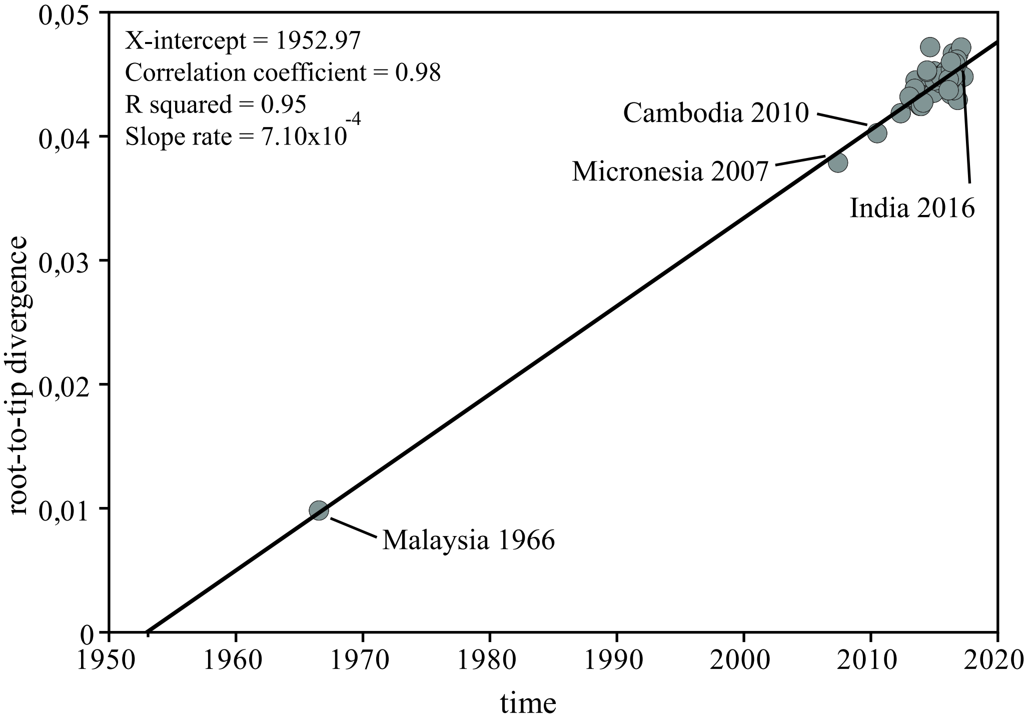

B

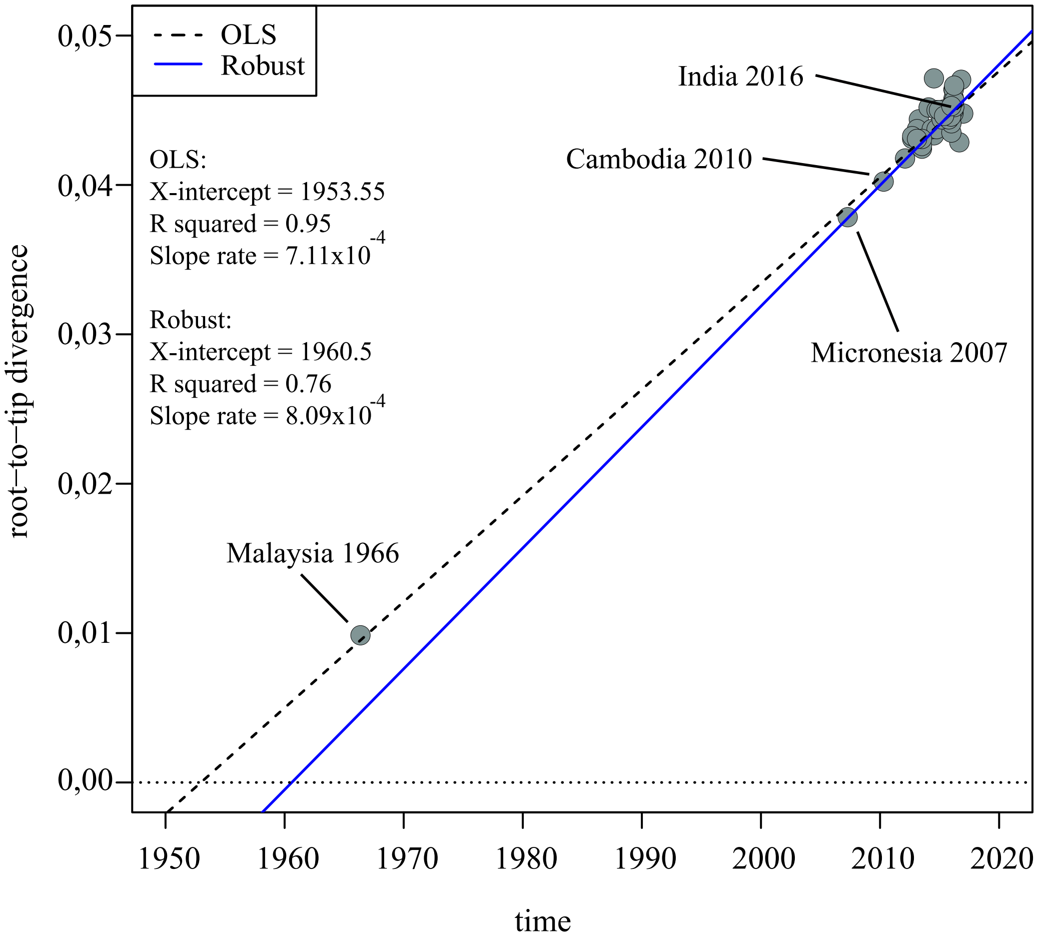

Supplement: Supplementary file 7 — Supplementary figure 7 [file 41426_2018_82_MOESM7_ESM.pdf]
