# Supplementary material for: Re-visiting the evolution, dispersal and epidemiology of Zika virus in Asia
Source: Emerg Microbes Infect. 2018 May 9;7:79. doi: 10.1038/s41426-018-0082-5 (PMC5940881; doi:10.1038/s41426-018-0082-5)
Supplement: Supplementary file 8 — Supplementary table 1 [file 41426_2018_82_MOESM8_ESM.pdf]

Supplementary table 1. Detailed summary of all diversification events summarized in Figure 1 as estimated from the BEAST analysis including 84 partial and complete Asian ZIKV genomes

**Node**	**Diversification event**	**tMRCA**	**95% HPD**	
**A**	Malaysia 1966 / other ZIKV	1954–04	1948–08	1959–05
**B**	India / other ZIKV	1956–10	1949–09	1964–05
**C**	Philippine–Micronesia / other ZIKV	1997–01	1994–09	1999–03
**D**	Cambodia / other ZIKV	2001–01	1999–02	2003–12
**E**	Vietnam / other ZIKV	2004–06	2002–06	2006–04
**F**	Thailand–Bangladesh / other ZIKV	2005–01	2003–11	2009–05
**G**	Indonesia / other ZIKV	2006–03	2004–09	2007–06
**H**	Thailand / other ZIKV	2006–09	2005–05	2007–01
**I**	Thailand / other ZIKV	2007–10	2006–06	2009–01
**J**	Thailand–Singapore / other ZIKV	2009–01	2007–12	2010–02
**K**	Vietnam / other ZIKV	2010–02	2009–01	2011–03
**L**	Vietnam / other ZIKV	2011–05	2010–01	2012–07
**M1**	Pacific islands / Pi–American ZIKV	2012–06	2011–12	2013–01
**M2**	Pacific islands / Pi–American ZIKV	2012–09	2012–01	2013–02
**M3**	Pacific islands / Pi–American ZIKV	2012–11	2012–07	2013–03
**M4**	Pacific islands / American ZIKV	2013–01	2012–09	2013–05
**N**	American ZIKV	2013–03	2012–11	2013–06
	**Mean evolutionary rate**	7.26×10^{-4}		
	**95% HPD evolutionary rate**	$6.28–8.19 \times 10^{-4}$		
