# Supplementary material for: Re-visiting the evolution, dispersal and epidemiology of Zika virus in Asia
Source: Emerg Microbes Infect. 2018 May 9;7:79. doi: 10.1038/s41426-018-0082-5 (PMC5940881; doi:10.1038/s41426-018-0082-5)
Supplement: Supplementary file 9 — Supplementary table 2 [file 41426_2018_82_MOESM9_ESM.pdf]

Supplementary table S2. Information on sequences used in the phylogenetic and dating analyses.

Name in trees	Genbank accession	Orgin	Collection date	Complete/Partial	If partial, which region	Coding nucleotide length
Mexico_KY606273|2016-06-30	KY606273	Mexico	2016-06-30	Complete	–	10272
Martinique_KU647676|2015-12	KU647676	Martinique	2015-12	Complete	–	10272
French_Guiana_KU758877|2015-12	KU758877	French Guiana	2015-12	Complete	–	10272
Peru_KY693678|2016-06-28	KY693678	Peru	2016-06-28	Complete	–	10272
Peru_KY693679|2016-07-11	KY693679	Peru	2016-07-11	Complete	–	10272
Suriname_KU312312|2015-10-02	KU312312	Suriname	2015-10-02	Complete	–	10272
Jamaica_KY785419|2016-06-13	KY785419	Jamaica	2016-06-13	Near complete	–	10030
Mexico_KY631493|2015-10-15	KY631493	Mexico	2015-10-15	Complete	–	10270
Puerto_Rico_KX601168|2015-12-01	KX601168	Puerto Rico	2015-12-01	Complete	–	10272
Honduras_KY693676|2016-08-26	KY693676	Honduras	2016-08-26	Complete	–	10267
Nicaragua_MF434522|2016-08-29	MF434522	Nicaragua	2016-08-29	Complete	–	10272
Panama_KX156776|2015-12-18	KX156776	Panama	2015-12-18	Complete	–	10272
Panama_KX156774|2015-12-18	KX156774	Panama	2015-12-18	Complete	–	10272
Colombia_MF574572|2015-12	MF574572	Colombia	2015-12	Complete	–	10272
Colombia_KU820897|2015-12	KU820897	Colombia	2015-12	Complete	–	10272
Brazil_KY558999|2016-07-10	KY558999	Brazil	2016-07-10	Complete	–	10271
Ecuador_KX879604|2016-04	KX879604	Ecuador	2016-04	Complete	–	10272
Venezuela_China_KU820898|2016-02-14	KU820898	Venezuela	2016-02-14	Complete	–	10272
Guatemala_KU501217|2015-11-01	KU501217	Guatemala	2015-11-01	Complete	–	10272
Brazil_KX197192|2015	KX197192	Brazil	2015	Complete	–	10272
Brazil_KY272991|2016-02-12	KY272991	Brazil	2016-02-12	Complete	–	10272
Honduras_KX694534|2015-01-06	KX694534	Honduras	2015-01-06	Complete	–	10272
Brazil_KX520666|2015-08	KX520666	Brazil	2015-08	Complete	–	10272
Brazil_KU729217|2015	KU729217	Brazil	2015	Complete	–	10272
Dominican_Republic_KY014300|2016-04-20	KY014300	Dominican Republic	2016-04-20	Complete	–	10272
USA_KY325476|2016-10-11	KY325476	USA	2016-10-11	Near complete	–	10234
USA_KY014323|2016-08-23	KY014323	USA	2016-08-23	Complete	–	10272
Cuba_MF159531|2017-04-19	MF159531	Cuba	2017-04-19	Complete	–	10270
Guadeloupe_KX673530|2016-04-21	KX673530	Guadeloupe	2016-04-21	Complete	–	10272
Guatemala_KU501216|2015-12-01	KU501216	Guatemala	2015-12-01	Complete	–	10272
Ecuador_KX879603|2016-04	KX879603	Ecuador	2016-04	Complete	–	10272
Cuba_MF438286|2017-02-12	MF438286	Cuba	2017-02-12	Complete	–	10271
Haiti_KY415988|2014-06-05	KY415988	Haiti	2014-06-05	Complete	–	10272
Haiti_KY415990|2014-06-02	KY415990	Haiti	2014-06-02	Complete	–	10272
Haiti_KU509998|2014-12-12	KU509998	Haiti	2014-12-12	Complete	–	10272
Haiti_USA_KX051563|2016-02-05	KX051563	Haiti	2016-02-05	Complete	–	10272
French_Polynesia_KX447518|2013-12	KX447518	French Polynesia	2013-12	Near complete	–	9062

French_Polynesia_KX447510|2013-12	KX447510	French Polynesia	2013-12	Complete	–	10272
French_Polynesia_KX447509|2013-12	KX447509	French Polynesia	2013-12	Complete	–	10272
French_Polynesia_KX447513|2013-12	KX447513	French Polynesia	2013-12	Complete	–	10272
French_Polynesia_KJ776791|2013-11-28	KJ776791	French Polynesia	2013-11-28	Complete	–	10272
French_Polynesia_KX447512|2013-12	KX447512	French Polynesia	2013-12	Complete	–	10272
French_Polynesia_KX447521|2014-02	KX447521	French Polynesia	2014-02	Near complete	–	8286
Tonga_exAustralia_KX806557|2016-02	KX806557	Tonga	2016-02	Complete	–	10272
Fiji_exJapan_LC191864|2016-04-21	LC191864	Fiji	2016-04-21	Complete	–	10272
American_Samoa_exChina_KU866423|2016	KU866423	American Samoa	2016	Complete	–	10272
American_Samoa_exChina_KX185891|2016-02-17	KX185891	American Samoa	2016-02-17	Complete	–	10272
French_Polynesia_KX447519|2013-11	KX447519	French Polynesia	2013-11	Near complete	–	8993
French_Polynesia_KX447520|2014-01	KX447520	French Polynesia	2014-01	Near complete	–	8864
French_Polynesia_KX447511|2014-01	KX447511	French Polynesia	2014-01	Complete	–	10272
French_Polynesia_KX369547|2013-10-25	KX369547	French Polynesia	2013-10-25	Complete	–	10272
French_Polynesia_KX447515|2013-11	KX447515	French Polynesia	2013-11	Complete	–	10272
French_Polynesia_KX447514|2014-01	KX447514	French Polynesia	2014-01	Complete	–	10272
French_Polynesia_KX447516|2014-01	KX447516	French Polynesia	2014-01	Complete	–	10272
French_Polynesia_KX447517|2014-01	KX447517	French Polynesia	2014-01	Complete	–	10272
Viet_Nam|KY131441|2013-01	KY131441	VietNam	2013-01	Partial	Envelope	1490
Viet_Nam|KY131442|2013-06	KY131442	VietNam	2013-06	Partial	Envelope	1512
VietNam_exJapan_LC219720|2016-11-22	LC219720	VietNam	2016-11-22	Complete	–	10272
Thailand_exTaiwan_KY126349|2016-10	KY126349	Thailand	2016-10	Partial	Capsid, PrM, Envelope	2382
Thailand_MF692778.1|2016-10	MF692778	Thailand	2016-10	Complete	–	10272
Singapore_KX827309|2016-08-28	KX827309	Singapore	2016-08-28	Complete	–	10272
Singapore_KX813683|2016-08-27	KX813683	Singapore	2016-08-27	Complete	–	10272
Singapore_KY241691|2016-09-13	KY241691	Singapore	2016-09-13	Complete	–	10271
Thailand_exTaiwan_KY126351|2016-05	KY126351	Thailand	2016-05	Complete	–	10272
Thailand_KU681081|2014-07-19	KU681081	Thailand	2014-07-19	Complete	–	10272
Thailand_KX051560|2013-07-09	KX051560	Thailand	2013-07-09	Complete	–	10272
Indonesia_exTaiwan_KY126345|2016-06	KY126345	Indonesia	2016-06	Partial	Capsid, PrM, Envelope	2382
Indonesia_KU179098|2014-12-30	KU179098	Indonesia	2014-12-30	Complete	–	10272
Malaysia_exTaiwan_KY126348|2016-10	KY126348	Malaysia	2016-10	Partial	Capsid, PrM, Envelope	2382
Thailand_KX051562|2015-01-16	KX051562	Thailand	2015-01-16	Complete	–	10270
Bangladesh_exChina_KY328290|2016-11-03	KY328290	Bangladesh	2016-11-03	Complete	–	10272
Thailand_KX051561|2013-09-21	KX051561	Thailand	2013-09-21	Complete	–	10271
Bangladesh|KY064008|2014-09-01	KY064008	Bangladesh	2014-09-01	Partial	PrM, envelope	630
Thailand_KX694532|2013-02-12	KX694532	Thailand	2013-02-12	Complete	–	10272
Thailand_Canada_KF993678|2013-02-19	KF993678	Thailand	2013-02-19	Near complete	–	10141
Thailand_KY272987|2016-08-30	KY272987	Thailand	2016-08-30	Complete	–	10272

VietNam_exTaiwan_KY126347|2016-09	KY126347	VietNam	2016-09	Partial	Capsid, PrM, Envelope	2382
Cambodia_JN860885|2010	JN860885	Cambodia	2010	Complete	–	10267
Philippines_KY003152|2016-09-01	KY003152	Philippines	2016-09-01	Partial	Envelope	1862
Philippines_KU681082|2012-05-09	KU681082	Philippines	2012-05-09	Complete	–	10272
Philippines_South_Korea_KY553111|2016-04	KY553111	Philippines	2016-04	Complete	–	10260
Micronesia_EU545988|2007-06-01	EU545988	Micronesia	2007-06-01	Complete	–	10269
India_MF173409.MF173410.MF173411|2016-11-14	MF173409; MF173410; MF173411	India	2016-11-14	Partial	capsid, envelope and NS2b/NS3	453, 773, 1393
Malaysia_KX601167|1966-07-14	KX601167	Malaysia	1966-07-14	Complete	–	10272
Uganda_MR766_NC_012532.1_1947	NC_012532.1	Uganda	1947	Complete	–	10259
Uganda_MR766_AY632535.2_1947	AY632535.2	Uganda	1947	Complete	–	10259
Uganda_MR766_KU963573.1_1947-04-20	KU963573.1	Uganda	1947-04-20	Complete	–	10260
Central_African_Republic_KF268949.1_1980-08	KF268949.1	Central African Republic	1980-08	Complete	–	10254
Central_African_Republic_KF268948.1_1979-11	KF268948.1	Central African Republic	1979-11	Complete	–	10269
